# Thyroid Gland Diffuse Lipomatosis: A Case Study and Comprehensive Literature Review

**DOI:** 10.3390/jcm13216376

**Published:** 2024-10-24

**Authors:** Angeliki Emmanouilidou, Michail Karanikas, Kalliopi Pazaitou-Panayiotou, Nickos Michalopoulos

**Affiliations:** 1 School of Medicine, Aristotle University of Thessaloniki, 54124 Thessaloniki, Greece; 2 Department of Endocrine Surgery, Genesis Hospital, 54301 Thessaloniki, Greece; michaelkaranikas@yahoo.gr (M.K.); nickos.michalopoulos@gmail.com (N.M.); 3 Division of Endocrinology, European Interbalkan Medical Center, 55535 Thessaloniki, Greece; kpazaitoupanayiotou@gmail.com

**Keywords:** diffuse lipomatosis, thyroid, amyloid goiter, case report, review

## Abstract

Diffuse lipomatosis of the thyroid (DLT) is an uncommon condition where mature fat cells infiltrate the thyroid gland, disrupting its normal structure. Although rare, it typically manifests as neck enlargement or symptoms of compression, including breathing difficulties, trouble swallowing, and voice changes, which can complicate diagnosis. This report presents a case of a 61-year-old woman with DLT, who exhibited a multinodular goiter and progressive neck swelling, and examines 53 additional cases from the existing literature. The analysis indicates that DLT is frequently misdiagnosed because of its similarities with other thyroid disorders. The precise mechanism underlying its development remains uncertain, but theories include oxygen deprivation in tissues, developmental abnormalities, and disruptions in fat metabolism. Surgical removal is the preferred treatment, especially for patients experiencing symptoms, and has shown favorable long-term outcomes. Additional studies should aim to elucidate the exact cause of DLT and enhance diagnostic precision, particularly in distinguishing it from other fat-containing thyroid lesions such as amyloid goiter and adenolipomas. A deeper understanding of this condition will inform better treatment approaches and enhance patient outcomes for this uncommon but significant thyroid disorder.

## 1. Introduction

Diffuse lipomatosis of the thyroid or DLT is characterized by infiltration of the parenchyma of the gland and replacement of the otherwise typical anatomical structure with mature adipose cells. It is not commonly found among other pathological conditions of the thyroid but nonetheless presents an interesting phenomenon. Although the thyroid is closely connected to other mesodermal structures during embryogenesis, contributing to fat deposition in areas around the cervical blood vessels in the subcapsular region of the anterior thyroid region, the presence of significant amounts of adipocytes within the thyroid stroma is rare [1,2,3,4]. Diffuse adipose tissue causing enlargement is often found in the thymus, parathyroid glands, pancreas, and salivary glands, as a result of atrophy-related parenchymal fatty metaplasia, whereas thyrolipoma is the most common condition in the thyroid [5,6,7]. Adenolipomas of the thyroid or thyrolipomas are well-circumscribed admixtures of adipose and follicular cells surrounded by a fibrous capsule consisting of a mixed neoplasm that is mesenchymo-epithelial [4]. Both thyrolipomas and diffuse lipomatosis are benign lesions of the gland, with the latter being more frequently associated with escalating growth clinically manifesting with compression symptoms from nearby organs such as dyspnea, hoarseness, dysphagia, and overall swelling. The enlargement observed in acquired and congenital goiters may also be caused by fat invasion rather than follicular proliferation [8,9]. The exact pathophysiological mechanism responsible for the diffuse nature of the fatty infiltration of the thyroid remains unclear. Here, we present an indigenous case and review the literature on DLT.

## 2. Case Report

**Figure 1:**
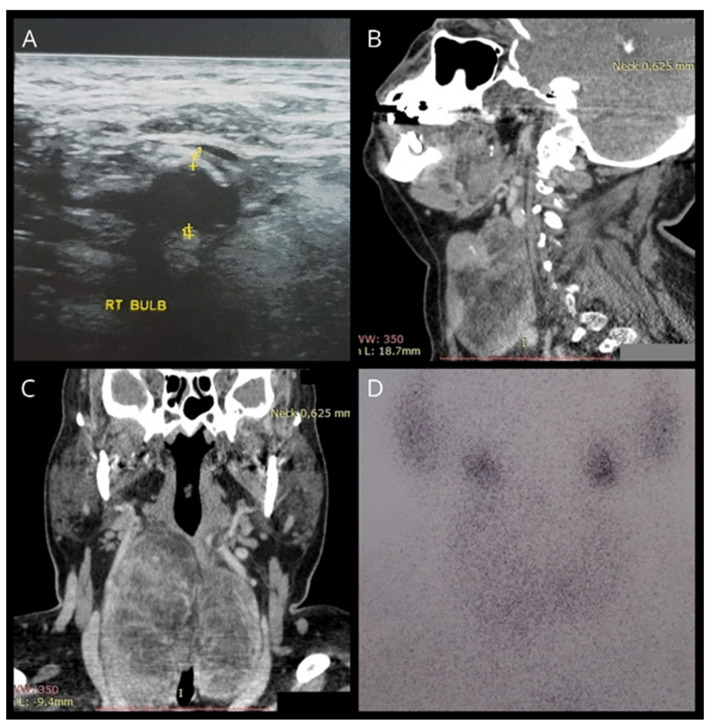
(**A**) Ultrasonography showing a hyperechogenic thyroid gland. (**B**,**C**) CT findings indicating a diffusely enlarged thyroid with hypodense areas and intrathoracic extensions. (**D**) Tc^99m^ scintigraphy showing heterogeneous uptake of the isotope with diffuse hypofixation.

**Figure 2:**
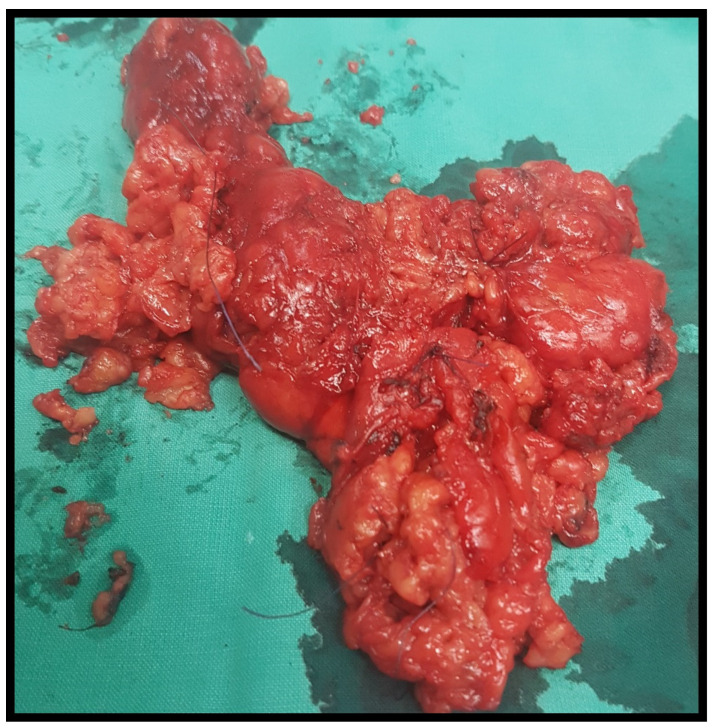
Thyroid specimen after total thyroidectomy.

**Figure 3:**
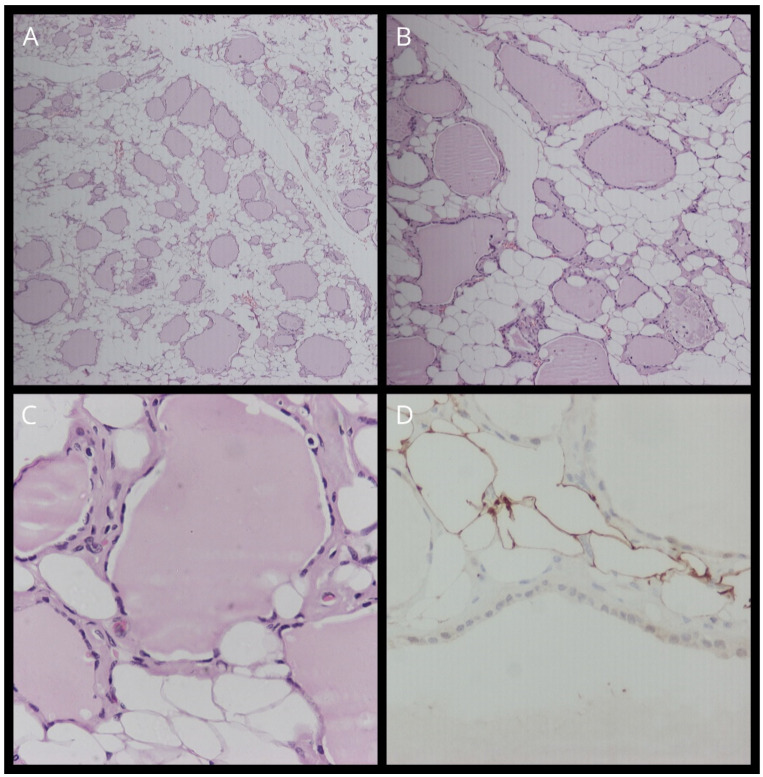
(**A**) Staining with hematoxylin–eosin (H&E) (magnification, ×10). (**B**) Staining with hematoxylin–eosin (H&E) (magnification, ×40). (**C**) Staining with hematoxylin–eosin (H&E) (magnification, ×100). (**D**) TTF-1 staining.

Our case involves a 61-year-old woman who was referred to our department for a total thyroidectomy. She had been diagnosed with a substernal multinodular goiter two years previously, and in the last six months, she gradually developed neck swelling, dyspnea, and dysphagia. Her medical history showed that she had chronic renal failure of unknown etiology, and she had been undergoing hemodialysis for the last year. She was also diagnosed with hypertension, diabetes mellitus, and obesity [BMI (body mass index): 37]. Upon clinical examination, a substantial thyroid mass with a soft consistency was palpated. Her thyroid function was normal [TSH (Thyroid Stimulating Hormone): 0.46 pmol/L, FT4 (Free Thyroxine): 20.09 pmol/L, FT3 (Free Triiodothyronine): 3.1 pmol/L)] and she tested negative for serum thyroperoxidase (anti-TPO) and thyroglobulin antibodies (anti-TG). Ultrasonography findings indicated an enlarged thyroid gland with dispersed hyperechoic regions. In accordance, CT (computed tomography) confirmed the presence of an enlarged gland showing low attenuation and septations. Computed tomography revealed that the thyroid had invaded the entire cervical area and the upper mediastinum, compressing the surrounding tissue (Figure 1). Thyroid scintigraphy with Tc^99m^ (Technetium-99m) revealed low uptake of radioactivity in the entire gland, suggesting the possibility of subacute thyroiditis. The patient underwent total thyroidectomy. The operation lasted for 2 h and proved to be an arduous challenge. Intraoperatively, the thyroid was friable upon touch, its capsule was very thin, and the gland was soft–fatty in texture, similar to a lipoma (Figure 2). Following surgery, the patient experienced no complications and was released two days later. Histopathological examination revealed fatty infiltration of the thyroid gland and degeneration of follicles. Immunohistochemical staining for thyroglobulin (TG) and thyroid transcription factor-1 (TTF1) verified the existence of a few colloid-filled thyroid follicles, whereas positive S100 staining demonstrated the preponderance of adipose tissue within the gland. (Figure 3). Therefore, the patient was diagnosed with diffuse thyroid lipomatosis. Two years after surgical treatment, the patient died because of complications associated with kidney failure.

## 3. Review

The first description of DLT in the medical literature was by Dhayagude [10] in 1942. Since then, we identified another 53 cases of thyroid lipomatosis including our own. We systematically researched MEDLINE, Embase, Scopus, and Web of Science for all articles involving thyroid lipomatosis cases. We included only reports where the gland was almost entirely and diffusely infiltrated by fat. Based on our criteria, we identified common characteristics among the above reports including sex, age, symptoms, thyroid function, renal status, initial diagnosis, treatment, histopathological features, and follow-up. Nine of the patients (16%) were described by Ge [1], two in 2016 by Bell [11], and, more recently, three cases by Celik [8]. The average age was 50 years (range, 11–78 years), and there was no significant sex predilection between males (47%) and females (53%). The most significant clinical feature was local compression (60%), which involved dysphagia, dysphonia/hoarseness, dyspnea, and neck swelling (46.7%), whereas one-third of the patients did not show any symptoms. Thyroid function was uncompromised in most patients (66%), and only a few patients had hypothyroidism (12.7%) or hyperthyroidism (21.3%). What appeared to be of notice was the fact that 40% of patients suffered from renal failure, with most of them being affected by secondary amyloidosis. Amyloid goiters are found more frequently in secondary amyloidosis than in primary amyloidosis and are commonly misdiagnosed as thyroid carcinoma [12]. In terms of diagnostic imaging, the most frequently employed techniques were thyroid ultrasound—U/S (72%), CT scans (67.4%), scintigraphy (23.2%), and magnetic resonance imaging—MRI (16.2%). Many patients were initially diagnosed with goiters (64.6%), either diffuse or multinodular, and one patient reported an amyloid goiter (2.1%), while a diagnosis of DLT was recently proposed in eight reports (16.7%). Among the reports that mentioned a treatment method, total thyroidectomy was the predominant choice (47.9%). Only one patient with a preliminary diagnosis of DLT was discharged without treatment, and partial thyroidectomy was an alternative, especially in patients with unilateral nodular disease. The average thyroid weight was 232.16 g (range 15–700 g). All cases revealed that the thyroid was almost entirely composed of mature adipose cells, and in cases of amyloid infiltration, the confirmation consisted of positive Congo red or thioflavin staining. Fat deposits that vary in amount are frequently found in amyloid goiter [7]. Amyloid infiltration was observed in almost half of patients (44.8%). We only included reports in which the gland was almost completely or in most parts infiltrated by fat tissue regardless of amyloid deposition, fibrosis, or lymphocyte aggregates. Only a minority of reports (43.4%) included follow-up information, and most (78.3%) had an uncomplicated post-operative course ranging from 1 day to 12 months. One patient reported respiratory difficulty 3 years after the first evaluation, three others died of complications, two because of renal insufficiency and the other after developing metastases in the residual thyroid lobe. Finally, a DLT case with concurrent secondary amyloidosis was revealed via the patient’s autopsy. Table 1 summarizes the characteristics of the DLT in these patients (Table 1).

## 4. Discussion

Numerous attempts have been made to explain the etiology of fatty infiltration in the thyroid gland. According to Dhayagude [10], fat deposits in colloid goiters may arise from the degeneration of follicular tissue due to damage such as hemorrhage, fibrosis, infarction, calcification, or cystic degeneration. In his textbook “*The borderland of Embryology and Pathology*”, Willis [53] describes an adenolipoma and explains that its presence could be a result of the metaplastic formation of fat. In amyloid goiters, it is presumed that adipose tissue is formed from stromal metaplasia of fibroblasts as a result of senile involution or tissue hypoxia [54]. Trites [55] hypothesized that some factors may have affected the primitive foregut during embryogenesis, causing the formation of mixed embryonic tumors and explaining fatty infiltration in congenital goiters. In various lipomas, it has been suggested that a disturbance in lipid metabolism could promote the accumulation of fat tissue [56], whereas others have argued that fat accumulation does not seem to depend on general factors such as obesity [57]. New research suggests that individuals with a BMI exceeding 25 are more likely to develop steatosis and have increased fat deposits between follicles in their thyroid glands. In certain instances, the amount of fat accumulation appeared to increase in proportion to the BMI value [58,59]. Obesity-related fat accumulation can be detected through the use of HAM56 (macrophages monoclonal antibody 56) immunostaining. This technique identifies macrophages that encircle senescent adipocytes, serving as a marker of late-stage obesity [60]. Chevsky et al. [14] proposed that adipose tissue might be incorporated into the thyroid during embryogenesis, along with striated muscle, before the gland capsule is formed. Schroder [4] postulated that DLT is associated with “displaced nests of embryonic structures, calling the entity “choristomatous adiposity”. A recent theory that attempts to explain the pathophysiology of the disease suggests that somatic mutations leading to the loss of succinate dehydrogenase-subunit B (SDHB) expression may play a role. Immunohistological staining revealed the loss of this protein in cells from DLT tissue, resulting in the deregulation of the mitochondrial respiration process and interference with lipid metabolism. This could result in a decrease in fatty acid oxidation, explaining the attenuation of fat in the gland and the replacement of normal follicles [6]. All of these theories could be plausible because they explain different aspects of infiltration, either regarding its diffuse presence in congenital and acquired goiters or the limitation of fatty deposition in certain regions of the gland.

Amyloidosis can either lead to or result from chronic kidney failure. The two main types of amyloidosis are primary, which stems from plasma cell disorders and is characterized by an increase in immunoglobulin light chains, and secondary (or reactive), which is linked to long-term inflammation, infections, and cancers. Both forms can potentially cause chronic renal failure due to amyloid accumulation. On the other hand, hemodialysis-associated amyloidosis, typically occurring after about ten years of treatment, is related to the ineffective filtration of β2 microglobulin during dialysis, leading to its buildup as Aβ2M amyloid protein. All these types of amyloidosis are connected to kidney failure. Although rare, the abnormal deposition of amyloids may also result in an amyloid goiter [61,62].

Imaging techniques are a common diagnostic tool in the investigation of diffuse goiter and are the presumed initial diagnosis in most cases. After extensive investigation, only nine reports assumed that the gland was infiltrated by fat tissue. Ultrasonographic findings most commonly indicate parenchymal heterogeneity [26], gland enlargement, and cystic or solid nodules with septations [25,28,41]. Computed tomography (CT) and fine-needle aspiration (FNA) biopsy are the gold standard for the early diagnosis of disease [25]. Tomographic findings consisted of an enlarged gland with low attenuation (−30 to −70 Hounsfield units) and heterogeneity in the parenchyma with a few areas showing hyperattenuation, probably indicating the presence of normal thyroid tissue [25,28,31,34,39]. In cases where compressing symptoms were present, invasion of the retropharyngeal space, expansion to the thoracic inlet, and compression of nearby organs such as the trachea or esophagus, were found [11,28]. Overall, almost all reports noted a low tomographic density of the thyroid stoma, but it was not low enough to be certain of fatty infiltration [11,34]. Lately, MRI (magnetic resonance imaging) has been more frequently used to confirm findings of low density in the stroma and increased signals with fat suppression in T1 and T2 sequences [40,44]. Scintigraphy with Tc^99m^ or I^123^ (Iodine-123) showed heterogeneity in radioactive uptake, occasionally indicating the presence of cold nodules [26,36]. Finally, FNA cytology or biopsy was performed in ambivalent cases expressing infiltration of the gland with fatty tissue, either in the background or predominantly. Fine needle aspiration is only reliable when samples are obtained from areas of fat infiltration. In other cases, the results can be highly variable, potentially suggesting anything from a colloid cyst to a possible follicular carcinoma [41,46,51]. However, clear guidelines for the definitive diagnosis of thyroid lipomatosis are eclipsed, and almost none of the above cases reached a diagnosis until after pathological findings were released [41]. A definitive diagnosis can be achieved after thyroidectomy if the pathological report describes diffuse gland infiltration [11].

The differential diagnoses include non-neoplastic fat-containing lesions such as adenolipoma, DLT, amyloid goiter, lymphocytic thyroiditis, heterotopic nests of adipose cells, parathyroid lipoma, or intrathyroid thymic tissue, and neoplastic lesions such as lipid-rich cell adenoma, liposarcoma encapsulated papillary carcinoma or anaplastic carcinoma. Adenolipoma can be easily differentiated from diffuse lipomatosis by its well-defined, encapsulated appearance along with the simultaneous admixture with proliferated thyroid follicles [25,26,40]. An amyloid goiter is usually found in systemic amyloidosis, either primary or secondary, and its pathognomonic characteristic of positive Congo red or crystal violet staining makes the diagnosis relatively straightforward [5,26]. However, there are cases where both amyloid and fat cell depositions were found in equal amounts or amyloid deposits were scarce amidst a thyroid stroma mainly composed of fat cells [26]. As there is no certainty regarding the criteria for the differential diagnosis of amyloid goiters with fatty infiltration from thyroid lipomatosis, we only considered cases where there was a clear predominance of fat tissue over amyloid proteins. Accumulation of various amounts of fat cells is a common finding in amyloid goiters because tissue hypoxia caused by a gradual increase in amyloid could drive fibroblasts to differentiate into fat cells, as previously stated [22]. Lymphocytic thyroiditis is associated with extensive infiltration of the stroma by lymphocytes, whereas heterotopic nests can be only found in the subcapsular regions of the gland [3,25,57]. The intimate embryologic origin of the thyroid with the parathyroid glands and thymus may be the cause of parathyroid lipomas, with characteristic cytoplasmic glycogen deposits, and ectopic thymic tissue [1,5]. Lipid-rich follicular adenomas can be distinguished by the presence of follicles with aggravated intracytoplasmic lipid formation, circular nuclei, and vesicular morphology [3,35]. Liposarcoma is a rare, aggressive neoplasm that usually expands rapidly beyond the thyroid capsule. Finally, there have been a few cases in which papillary carcinoma was found within diffusely enlarged goiters infiltrated with fat. Carcinomas can be identified via immunostaining for p53, CD68 (Cluster of Differentiation 68), HBME-1 (Human Bone Marrow Endothelium Marker-1), and CK19 (Cytokeratin 19) [63,64,65]. In one case, the histopathological features and immunohistochemical profile of the tumor were evident, and adipose cells were occasionally found in small amounts within the stroma [31].

The swift enlargement of the thyroid and its tendency to press on surrounding structures necessitates distinguishing it from anaplastic cancer and Riedel’s thyroiditis. In most instances, imaging techniques can differentiate between lipomatosis and anaplastic cancer, guiding appropriate diagnosis and treatment strategies [31]. It is noteworthy that invasive fibrous thyroiditis, though rare, should be considered, particularly as it shares alarming symptoms like difficulty swallowing and breathing, and exhibits similar CT findings. Nevertheless, supplementary imaging methods such as ultrasound and MRI can typically discern between these conditions, as Riedel’s thyroiditis presents hypoechoic and low signal density characteristics due to fibrotic tissue infiltration. While FNA biopsy or cytology can probably differentiate thyroid lipomatosis from the aforementioned conditions, only an open surgical biopsy can definitively distinguish between anaplastic carcinoma and Riedel’s thyroiditis [66,67,68].

A distinguishing feature of diffuse thyroid lipomatosis is the infiltration and replacement of what would be an otherwise normal thyroid stroma by mature adipose cells [33]. Macroscopically, the gland has a pale yellow-brown color and a soft and friable texture. In most cases, the gland size exceeded the normal weight (10–20 g), the lobes were enlarged, and when cut, they had a lobular appearance [5,11,31]. Schroder et al. [4] noted that the gland may resemble a congenital goiter because of its progressive growth during the first decade of life. Microscopic analysis revealed that mature adipocytes lacking encapsulation had replaced normal thyroid follicles [5,11,42,54]. The fat tissue is lobulated by strands of fibrous tissue. The remaining indigenous cells were clustered in random amounts and were scattered throughout the fatty stroma [54]. The follicles were lined with cuboidal epithelium and exhibited colloid accumulation [31]. Infiltration of fat with scarce lymphocytes was observed in a few cases, whereas the deposition of pink unshaped material was found around the remnants of the follicular tissue [5,11]. Only in instances of systemic amyloidosis was this discovery described; in these cases, the amyloid A protein was identified through immunohistochemical staining and validated by apple-green birefringence during polarized microscopy using Congo red staining [11]. Papillary carcinoma coincided with DLT in three cases and was described as a localized group of follicles surrounded by fat, with oval cells and enlarged ground glass nuclei. Immunohistochemical positivity for thyroglobulin and TTF-1 confirmed the diagnosis [3,69].

Total thyroidectomy via a transverse horizontal cervical incision appears to be an appropriate therapeutic option for patients with symptomatic swelling [35]. Caution should be exercised in view of the softness and friability of the thyroid gland to avoid extensive intra-operative bleeding. Mobilization of the gland should be performed meticulously, with minimal traction during detachment and identification of the adjacent laryngeal nerves and parathyroid glands [25]. Hemithyroidectomy was also performed on some occasions where the findings could not exclude malignancy. A subsequent histopathological evaluation confirmed the diagnosis of diffuse thyroid lipomatosis. Biopsy samples may have been obtained from areas with scarce or no fat content [42]. Our review indicates that there should be a surgical attempt to assuage the patient only in cases in which compression symptoms are present. If the patient is asymptomatic and DLT is confirmed via imaging techniques or biopsy, the patient should be discharged and follow-up should be advised in the next few months to re-estimate the extent of the disease [40]. Owing to the underlying pathophysiological mechanisms of the disease, in cases where DLT is confirmed before or during surgery, the potential presence of ectopic thyroid tissue should be investigated [47]. Finally, in cases with thyroid swelling and hyperactivity indicated by scintigraphy, a CT scan should be ordered to examine the consistency of the gland. In the case of a possible DLT diagnosis, radioiodine ablation therapy can be dismissed altogether and replaced directly by total thyroidectomy [34].

## 5. Conclusions

Diffuse lipomatosis of the thyroid gland is a rare but fascinating condition that involves the replacement of normal thyroid tissue with fatty infiltration. Our review of the literature and presented case report highlight the clinical features, diagnostic challenges, and treatment options associated with this condition. Our results indicate that surgical intervention should be considered in symptomatic patients as it has been shown to lead to positive long-term results in most cases. Further research is needed to better understand the underlying mechanisms of DLT, which will enable the development of more accurate diagnostic and therapeutic approaches and improve patient outcomes.

## Figures and Tables

**Table 1:** Summary of thyroid lipomatosis case reports.

Reference	Sex/Age	Clinical Features	Thyroid Function	Renal Failure	Imaging Findings	Initial Diagnosis	Treatment	Thyroid Size/Weight	Pathology	Amyloid Deposition	Follow-Up
[10]	M/32	Local compression	NS (Not Stated)	No	NS	Diffuse goiter	Excision	10 × 8 × 5.5 cm (R), 8 × 6 × 5.5 cm (L)/500 g	Diffuse infiltration of fibrous-fatty tissue, anomalous follicles, focal fibrosis	NS	NS
[13]	F/11	No	BMR (basic metabolic rate) +3% (hyperthyroidism)	No	NS	Diffuse goiter (since birth)	Excision	×5 normal thyroid size	Diffuse fat infiltration alongside small thyroid follicles and lobules with fibrous septa	NS	NS
[14]	M/15	No	Normal	No	NS	Diffuse goiter (since birth)	Excision	12 × 7 × 4 cm (R), 8 × 5 × 2.5 cm (L)/253 g	Diffuse fat infiltration, small and medium thyroid follicles, stromal edema	NS	NS
[15]	M/58	Local compression	Hyperthyroidism	No	NS	Diffuse goiter	Excision	NS	Diffuse fat infiltration surrounding normal follicles, stromal fibrosis, lymphocyte aggregation	NS	NS
[16]	F/51	No	Normal	No	NS	Diffuse goiter	Excision	NS	Diffuse fat infiltration surrounding normal follicles, stromal fibrosis	NS	NS
[17]	F/73	No	Normal	No	NS	Diffuse goiter	Excision	NS/120 g	Diffuse fat infiltration around normal follicles, lymphocyte aggregation	NS	NS
[18]	M/12	No	Normal	No	NS	Right neck mass	Excision	13 × 8 × 6.5 cm (R)/415 g	Diffuse fat infiltration surrounding normal thyroid follicles, presence of lymphocytes and fibrous tissue	NS	NS
[19]	M/26	Neck swelling, hoarseness, dysphagia	Normal	Yes	CT: substantial mass exerting pressure in the front and side areas.	Cervical mass	Total thyroidectomy	NS	Diffuse infiltration of thyroid by fat	Yes	NS
[20]	F/77	No	Normal	No	U/S: hyperechoic mass. CT: iso- and hypodense mass with mild contrast enhancement.	Cervical mass	Excision	NS/700 g	Thyroid follicles surrounded by adipose cells	NS	NS
[21]	M/38	Neck swelling	Normal	No	Tc^99m^ scintigraphy: widespread bilateral enlargement and an area of reduced uptake in the left lobe.	Diffuse enlargement of thyroid	Excision	4 × 3 × 2 cm (I), 13 × 7 × 3 cm (R), 15 × 9 × 5 cm (L)/465 g	Mature adipose tissue surrounded the different sized but otherwise normal follicles of the thyroid	No	Well—post-operatively
[22]	F/58	Neck swelling, dysphagia, hoarseness, hyperthermia	Hypothyroidism	Yes	U/S: widespread enlargement of the thyroid with varied tissue density. Scintigraphy: overgrowth of the gland with uneven distribution of radioactive uptake.	Diffuse goiter	Total thyroidectomy	9.5 × 7 × 5 cm (R), 8 × 6 × 3.5 cm (L)/230 g	Diffuse fatty infiltration, loss of most thyroid follicles, eosinophilic substance deposition in the hyalinized stroma, lymphocytic thyroiditis	Yes	NS
[23]	M/64	Enlarged thyroid, dyspnea	Normal	Yes	U/S: heterogeneous diffuse goiter. CT: hypodense (fat-like) goiter, compressing the nearby airway. Scintigraphy: gland enlargement with uneven uptake.	Diffuse goiter	NS	NS	NS	NS	NS
[24]	M/45	Neck swelling, respiratory distress	Normal	No	CT: enlarged thyroid, with no retrosternal element, compressing the airway.	Diffuse thyroid enlargement	Subtotal thyroidectomy	2 × 1.5 × 1 cm (I), 9 × 7 × 4 cm (R), 9 × 5 × 3.5 cm (L), 4 × 3 × 2 (P)/225 g	Colloid filled thyroid follicles, fatty infiltration of interfollicular stroma	No	NS
[1]	F/67	Dysphagia	Normal	Yes	Scintigraphy: hypofunctioning nodule on the left lobe.	Enlarged nodular left thyroid lobe	Left thyroidectomy	7 × 3.5 × 2 cm (L)/41 g	Thyroid follicles separated by fat, adipose tissue density varied from 30 to 90% of total tissue, fat infiltration in adenomatous nodules	No	Well—2 years after
[1]	F/59	No	Hypothyroidism	No	Tc^99m^ scintigraphy: hot nodule in the right lobe. U/S: 1 year later revealed hypo and isoechoic areas in left lobe.	Right thyroid mass	Right thyroid lobectomy and isthmusectomy/left thyroidectomy	7 × 4 × 1.2 cm (R+I), 6 × 3 × 1.2 cm (L)/56 g	Nodular and diffuse pattern of fatty infiltration, presence of a 0.8 cm papillary carcinoma	No	Well—6 years after
[25]	F/40	No	Hyperthyroidism	No	U/S: several nodules within the thyroid gland, with a subset of these nodules exhibiting cystic components.	Multinodular goiter	Excision	NS	Colloid filled thyroid follicles of various sizes, replacement of stroma by mature adipose tissue	No	Well—post-operatively
[9]	M/37	Neck swelling, dyspnea	Normal	Yes	U/S: homogeneous, enlarged thyroid gland with a calcified, hyperechoic nodule in the insthmus. CT: confirmed the enlargement.	Diffuse thyroid enlargement	Total thyroidectomy	NS/304 g	Widespread deposition of eosinophilic material with scarcity of thyroid follicles, diffuse lipomatosis and amyloidosis (secondary)	Yes	Well—third post-operative day
[5]	M/62	Neck swelling, dyspnea	Normal	No	U/S: diffuse enlargement of thyroid.	Nodular goiter	Near-total thyroidectomy	12 × 10 × 6 cm (R), 15 × 8 × 6 cm (L)/285 g	Colloid filled thyroid follicles, diffuse infiltration of stroma with mature adipose tissue, scarce lymphocytic infiltration	No	NS
[26]	M/43	Neck swelling, dyspnea	Normal	Yes	U/S: heterogeneous thyroid. Tc^99m^ scintigraphy: diffuse uptake and a cold area in left lobe.	Enlarged thyroid	Total thyroidectomy	6 × 2 × 1 cm (I), 7.5 × 6 × 4.5 cm (R), 6.5 × 4.5 × 2 (L)/160 g	Scattered thyroid follicles in a dense mature fat stroma	Yes	NS
[27]	M/55	Neck swelling, cough	Normal	Yes	U/S: enlarged, hyperechoic thyroid gland. CT: thyroid enlargement with a soft tissue exceeding that of fat, resulting in tracheal displacement.	Diffuse goiter	Total thyroidectomy	4 × 2 × 1 cm (I), 9 × 6 × 4 cm (R), 7 × 5 × 2.5 cm (L)/148 g	Infiltration of thyroid by adipose tissue, small amounts of colloid filled follicles remained	Yes	Well—post-operatively
[28]	F/52	No	Normal	NS	U/S: enlarged hyper-ecogenic thyroid gland. CT: a large gland with fatty infiltration.	Diffuse lipomatosis	NS	NS	NS	NS	Difficulty breathing—3 years later
[29]	M/46	NS	Hypothyroidism	No	U/S: hypoechoic mass in the right thyroid lobe.	Cervical mass	Partial thyroidectomy (same procedure at 3 years of age)	NS	Infiltration of thyroid by mature adipocytes; same results as 43 years before	No	NS
[2]	F/32	Neck swelling	Hyperthyroidism	No	U/S: diffusely enlarged thyroid with mixed echogenicity. CT: heterogeneously enhanced gland with soft tissue density. Tc^99m^ scintigraphy: hyperfunctioning enlarge gland.	Toxic multinodular goiter	Total thyroidectomy	11 × 5 × 2 cm (total)/88 g	Thyroid follicles filled with colloid, diffuse fatty infiltration of stroma	No	NS
[3]	M/37	Neck swelling	NS	No	No	Nodular goiter	Total thyroidectomy	5 × 11 × 15 cm (total)/NS	Thyroid tissue replaced by mature adipocytes, papillary thyroid carcinoma	NS	NS
[6]	M/47	NS	Hypothyroidism	No	U/S: hypoechoic heterogeneous mass in the thyroid lobe. CT and MRI: identified the lesion and indicated fat infiltration of the gland.	Fatty infiltration of thyroid	Excision	16.5 × 8 × 5.5 cm (total)/250 g	Atrophic follicles of thyroid alongside mature adipose tissue infiltration, SDHB loss of expression (follicular or adipose cells)	No	NS
[8]	M/25	NS	Hypothyroidism	NS	NS	NS	NS	NS	Various sizes of thyroid follicles, abundant distribution of mature fat around thyroid tissue	NS	NS
[8]	F/19	NS	Hypothyroidism	NS	NS	NS	NS	NS	Various sizes of thyroid follicles, abundant distribution of mature fat around thyroid tissue	NS	NS
[8]	M/63	NS	Normal	NS	NS	NS	NS	NS	Preservation of a few thyroid follicles, abundant distribution of mature fat around follicles	NS	NS
[30]	M/69	Hoarseness, exertional dyspnea	NS	Yes	Solitary nodule exhibiting both solid and cystic components, accompanied by widespread lipomatous transformation of the surrounding tissue.	Diffuse fatty conversion and solid/cystic nodule of thyroid	Right hemithyroidectomy/completion thyroidectomy	NS	Diffuse infiltration of thyroid stroma by mature fat, insular carcinoma	Yes	NS
[31]	F/67	Neck swelling, respiratory distress	Hyperthyroidism	Yes	U/S: diffuse enlargement of thyroid. CT: heterogeneous goiter with hypodense nodules.	Diffuse goiter	Total thyroidectomy	2 × 2 cm (I), 12 × 6 × 3.5 cm (R), 10 × 5 × 2.5 cm (L)/215 g	Infiltration of thyroid by mature fat, scarcity of colloid follicles	No	Well— post-operatively
[11]	F/36	Dyspnea	Normal	Yes	U/S: cystic nodule in the right thyroid lobe and a hyperechoic one in the left. CT: diffuse hypodense enlarged gland, mildly displacing the trachea.	Multinodular goiter	Excision	9.8 × 9.5 × 4.5 cm (total)/144 g	Replacement of normal thyroid tissue by mature adipocytes except from small remnants of normal colloid filled follicles	Yes	NS
[32]	M/73	Neck swelling, dyspnea, hoarseness	Normal	No	U/S: diffuse enlargement of the thyroid with altered echogenicity and nodular echogenic lesions in both lobes. CT and MRI: fat accumulation in the gland.	Thyrolipomatosis	Near-total thyroidectomy	NS	Thyrolipomatosis	NS	NS
Present Study	F/61	Neck swelling, dyspnea, dysphagia	Normal	Yes	U/S: enlarged thyroid gland with hyperechoic areas. CT: large size of gland, diffuse low parenchymal density, and retrosternal extension. Tc^99m^ scintigraphy: low uptake diffusely.	Diffuse goiter	Total thyroidectomy	NS	Fatty infiltration of thyroid, degeneration of follicles	NS	Dead–after 3 years (because of kidney failure)
[33]	F/68	NS	Normal	No	CT: diffuse enlargement of the thyroid, airway compression and calcification bilaterally.	Diffuse goiter	Total thyroidectomy	NS	Fatty infiltration of thyroid, hyperplastic follicles alongside stromal sclerosis and calcification	No	Well—after 3 months
[34]	F/49	Neck swelling	Subclinical hyperthyroidism	No	CT: diffuse fatty infiltration of the thyroid. Scintigraphy: thyromegaly and low uptake of the gland.	Diffuse goiter	Radioiodine ablation (RAI)/total thyroidectomy	NS	Infiltration of thyroid stroma by fat	NS	Well—post-operatively
[35]	F/53	Local compression, dysphagia	Normal	Yes	CT: large heterogeneous thyroid, extending retrosternally and causing deviation of the airway.	Non-toxic diffuse multinodular goiter	Total thyroidectomy	2.5 × 2 × 1.5 cm (I), 5 × 2.5 × 2 cm (R), 11 × 7 × 3.5 cm (L)/415 g	Admixture of adipocytes with follicular cells	NS	NS
[36]	F/55	Neck swelling, hoarseness	Normal	No	U/S: heterogeneous diffuse goiter. Scintigraphy: heterogeneous uptake with cold and hot nodules.	Diffuse goiter	Total thyroidectomy	1.5 × 1 cm (I), 5.5 × 3.5 × 3 cm (R), 4 × 2.5 × 2.2 cm (L)/NS	Replacement of thyroid tissue by mature fat, few distended thyroid follicles	No	Well—post-operatively
[37]	F/48	Dysphagia	Subclinical hyperthyroidism	Yes	U/S: enlargement of the thyroid. CT and MRI: large size of the gland and the infiltration by fat.	Amyloid goiter	Total Thyroidectomy	13.5 × 4.5, 6.5 × 3.5 3.5 × 2.5 cm/NS	Adipose metaplasia of thyroid stroma	Yes	Well—post-operatively
[38]	M/20	Dysphagia	Normal	No	U/S and CT: large and heterogeneous left thyroid mass.	Follicular nodule	Left hemithyroidectomy	NS	Diffuse fatty infiltration of thyroid	NS	NS
[39]	M/73	Neck swelling, dyspnea, hoarseness	Normal	No	U/S: diffuse thyroid enlargement with multiple echogenic areas bilaterally. CT and MRI: fat accumulation in both lobes.	Diffuse lipomatosis of thyroid	Total thyroidectomy	NS	Diffuse presence of fat cells in the thyroid	No	NS
[40]	M/72	No	NS	Yes	U/S: thyroid enlargement with increasing echogenicity. CT and MRI: large, infiltrated by fat thyroid gland.	Diffuse lipomatosis of thyroid	No	NS	NS	NS	NS
[41]	M/46	Dyspnea, hoarseness, dysphagia	Normal	No	U/S: enlargement of thyroid with iso and hyporechogenic nodules. U/S—1 year later: revealed hyperechogenic nodules. CT: confirmed large size of gland and indicated parenchymal hypodensity.	Diffuse goiter	Total thyroidectomy	10.5 × 6.5 × 4.3 (R), 11.1 × 4 × 2.6 cm (L)/237 g	Normal follicular architecture, fat infiltration of stroma	Yes	Well—post-operatively
[42]	F/52	No	Normal	No	U/S: slight hypoechoic pseudo nodular area.	Goiter/suspicious nodule	Left hemithyroidectomy	6 × 4 × 2 cm (L)/26 g	Fatty infiltration of stroma, predominance of adipocytes in most regions	NS	Well—after 6 months
[43]	M/48	Neck swelling	Normal	Yes	U/S: heterogeneous multinodular goiter.	Multinodular goiter	Total thyroidectomy	NS	Diffuse and nodular infiltration of thyroid by adipose cells, scarce thyroid follicles	No	Well—after 12 months
[44]	M/40	Neck swelling	Normal	No	U/S: hyperechogenic thyroid nodule extending bilaterally. CT and MRI: confirmed fat lesion.	Thyrolipoma and thymolipoma	Total thyroidectomy	NS	Thyroid follicular cells surrounded by mature adipocytes	No	Well—after 2 weeks
[45]	F/69	Neck swelling, dyspnea, dysphagia, hoarseness	Normal	No	U/S: multinodular goiter with iso-hyperechoic nodules. CT: compression and deviation of trachea.	Multinodular goiter	Total thyroidectomy	1.5 × 1 × 1 cm (I), 6.5 × 5 × 3 cm (R), 5 × 3 × 2.5 cm (L)/NS	Diffuse infiltration of adipocytes and replacement of normal thyroid follicles	NS	Well—post-operatively
[46]	F/54	Neck swelling, hoarseness, dysphagia	Normal	Yes	U/S: enlarged thyroid with an isoechoic nodule. CT: verified thyroid enlargement, deviation of the trachea and presence of fat.	Diffuse lipomatosis of thyroid and follicular neoplasm	Total thyroidectomy	2.6 × 4.5 × 2 cm (I), 4.5 × 7.5 × 3.4 cm (R), 3.2 × 6.7 × 2.8 cm (L)/81 g	Papillary thyroid carcinoma, admixture of thyroid follicles with mature adipose tissue	Yes	Dead—after 5 months (urinary tract infection—sepsis)
[47]	F/57	Dyspnea, dysphagia, local compression	Hyperthyroidism	No	CT: enlarged thyroid compressing and deviating the trachea.	NS	Total thyroidectomy	NS	NS	NS	NS
[48]	F/60	NS	Hyperthyroidism	Yes	CT: enlarged, heterogeneous thyroid with low density lesions.	NS	Autopsy	NS	Replacement of normal thyroid follicles by diffuse fat deposits	Yes	Deceased
[49]	F/44	Tongue mass, dysphagia, weight loss	NS	No	U/S: multinodular goiter with a hypoechogenic nodule in the left lobe and bilateral lymphadenopathy. CT: diffuse thyromegaly and fatty infiltration.	Multinodular goiter	Right hemithyroidectomy	6 × 3.5 × 2 cm (R)/15 g	Diffuse fat metaplasia of stromal thyroid tissue indicative of thyrolipomatosis	No	Dead—after 5 months (septic shock after metastasis of squamous cell carcinoma of the tongue to the remaining left thyroid lobe)
[50]	F/78	Neck swelling, dysphagia, hoarseness	Normal	Yes	U/S: diffuse enlargement of thyroid with uniform echogenicity. CT: confirmed large size, fat density of the gland and tracheal deviation.	Multinodular goiter	Total thyroidectomy	22.4 × 8.5 × 5 cm/300 g	Infiltration of the gland by mature adipocytes, a few extended thyroid follicles and a stroma filled with adipocytes and fibrotic tissue	NS	Well—post-operatively
[51]	F/64	Weight loss, fatigue, enlarged thyroid	Hyperthyroidism	Yes	U/S and CT: fatty infiltration of thyroid.	Diffuse thyroid lipomatosis	Methimazole	NS	Benign adipose tissue with entrapped thyroid follicles (biopsy)	Yes	NS
[52]	F/51	NS	NS	Yes	MRI: enlarged and hypodense thyroid mass with retrosternal extension.	Multinodular goiter	Total thyroidectomy	9.4 × 6.6 × 5.7 cm (R), 8.5 × 5.2 × 4.7 cm (L), 2.2 × 1.2 × 1 cm (I)/NS	Lobules of adipocytes with few areas of atrophic thyroid follicles, papillary carcinoma	Yes	Well—after 6 months

## Data Availability

All relevant data are within this paper.

## References

[1] Ge Y., Luna M.A., Cowan D.F., Truong L.D., Ayala A.G. (2009). Thyrolipoma and thyrolipomatosis: 5 case reports and historical review of the literature. Ann. Diagn. Pathol..

[2] Sanuvada R., Chowhan A., Rukmangadha N., Patnayak R., Yootla M., Amancharla L. (2014). Thyrolipomatosis: An inquisitive rare entity. Gland. Surgery.

[3] Nandyala H.S., Madapuram S., Yadav M., Katamala S.K. (2015). Diffuse lipomatosis of the thyroid gland with papillary microcarcinoma: Report of a rare entity. Indian J. Pathol. Microbiol..

[4] Schroder S., Bocker W., Hüsselmann H., Dralle H. (1984). Adenolipoma (Thyrolipoma) of the Thyroid Gland Report of Two Cases and Review of Literature. Virchows Archiv A.

[5] Dombale V.D., Javalgi A.P. (2011). Symmetric Diffuse Lipomatosis of the Thyroid Gland. J. Clin. Diagn. Res..

[6] Lau E., Freitas P., Costa J., Batista R., Máximo V., Coelho R., Matos-Lima L., Eloy C., Carvalho D. (2015). Loss of mitochondrial SDHB expression: What is its role in diffuse thyroid lipomatosis?. Horm. Metab. Res..

[7] Daboin K.P., Ochoa-Perez V., Luna M.A. (2006). Adenolipomas of the head and neck: Analysis of 6 cases. Ann. Diagn. Pathol..

[8] Çelik Z.E. (2015). Mature Fat Containing Thyroid Lesions. Eur. J. Gen. Med..

[9] Citgez B., Uludag M., Yetkin G., Akgun I., Yener S., Polat N., Isgör A. (2011). Amyloid goiter with diffuse lipomatosis. World J. Endocr. Surg..

[10] Dhayagude R.G. (1942). Case report: Massive fatty infiltration in a colloid goiter. Arch. Pathol..

[11] Bell S., Sosa G.A., del Valle Jaen A., Russo Picasso M.F. (2016). Thyroid lipomatosis in a 36-year-old patient with rheumatoid arthritis and a kidney transplant. Endocrinol. Diabetes Metab. Case Rep..

[12] Munzinger U. (1974). Amyloid Goiter. Schweiz. Med. Wochenschr..

[13] Simard L.C. (1945). Une nouvelle forme de goître: La scléro-lympho-lipomatose thyroidiènne. Union. Med. Can..

[14] Chesky V.E., Dreese W.C., Hellwig C.A. (1953). Adenolipomatosis of the thyroid: A new type of goiter. Surgery.

[15] Bielicki F., Dawiskiba E., Kasprzak A., Kawecki K., Zagrobelny Z. (1968). [*Struma lipomatosa*]. Pol. Tyg. Lek..

[16] Dalforno S., Donna A. (1969). Lipomatosi diffusa della tiroide (*Struma lipomatose*). Cancro.

[17] Asirwatham J.E., Barcos M., Shimaoka K. (1979). Hamartomatous adiposity of thyroid gland. J. Med..

[18] Simha M.R., Doctor V.M. (1983). Adenolipomatosis of the thyroid gland. Indian J. Cancer.

[19] Téllez R., Le Cerf P., Araos F., Michaud P. (1996). Diffuse fatty infiltration of the thyroid gland associated to amyloidosis in a patient with chronic renal failure. Rev. Med. Chil..

[20] Paoletti H., Tourrette J., Terrier J., Colineau X., Thiebaut C., Dussaut J.P., Pujol A., Muyard B., Nun P., Solacroup J.C. (1997). [Diffuse thyroid lipomatosis]. J. Radiol..

[21] Arslan A., Lent Alíç B., Kemal Uzunlar A., Seyin Büyü H., Sarí I. (1999). Diffuse lipomatosis of thyroid gland. Auris Nasus Larynx.

[22] Himmetoglu C., Yamak S., Tezel G.G. (2007). Diffuse fatty infiltration in amyloid goiter. Pathol. Int..

[23] Di Scioscio V., Loffreda V., Feraco P., Luccaroni R., Palena L.M., Balbi T., Zompatori M. (2008). Diffuse lipomatosis of thyroid gland. J. Clin. Endocrinol. Metab..

[24] Gupta R., Arora R., Sharma A., Dinda A. (2009). Diffuse lipomatosis of the thyroid gland: A pathologic curiosity. Indian J. Pathol. Microbiol..

[25] Pradeep P.V., Kumar R., Ragavan M., Ramakrishna B.A. (2010). Diffuse lipomatosis of thyroid with hyperthyroidism. J. Postgrad. Med..

[26] Gonulalan G., Esen H., Mehmet E., Cakir M. (2012). Thyroid Lipomatosis. Intern. Med..

[27] Jacques T.A., Stearns M.P. (2013). Diffuse lipomatosis of the thyroid with amyloid deposition. J. Laryngol. Otol..

[28] Lo R., Donaldson C. (2013). Diffuse Lipomatosis of the Thyroid Gland. Ultrasound Q..

[29] Costa J., Pardal J., Máximo V., Gonçalves F., Eloy C. (2013). Diffuse lipomatosis of thyroid: A case report. Virchows Archiv..

[30] Liyanaarachchi N., Lim A., Donaldson E. (2016). Diffuse lipomatosis and amyloid deposition of the thyroid gland associated with poorly differentiated/insular carcinoma of the thyroid: Report of a rare entity. Pathology.

[31] Ben Gamra O., Romdhane N., Nefzaoui S., Mahjoubi M., Abid W., Koubaa W., Hariga I., Chadli A., Mbarek C.C. (2016). Diffuse lipomatosis of the thyroid gland. Egypt. J. Ear Nose Throat Allied Sci..

[32] Kumar R., Bhargava A., Jaiswal G. (2016). A Case Report on Radiologic Findings of Thyrolipomatosis: A Rare Fat Containing Lesion diffusely Infiltrating throughout the Thyroid Gland. J. Kathmandu Med. Coll..

[33] Ishida M., Kashu I., Morisaki T., Takenobu M., Moritani S., Uemura Y., Tsuta K. (2017). Thyrolipomatosis: A case report with review of the literature. Mol. Clin. Oncol..

[34] Harisankar C.N.B. (2018). A Rare Case of Thyrolipomatosis Presenting with Latent Hyperthyroidism. Indian J. Nucl. Med..

[35] Hijazi D.M., Addas F.A., Alghanmi N.M., Marzouki H.Z., Merdad M.A. (2018). An enlarged goiter presenting with a rare diffuse lipomatosis of the thyroid gland. Am. J. Case Rep..

[36] Ahmed J., El Amine R., Bouziane C. (2018). Diffuse Lipomatosis of Thyroid—Case Report. Surg. Sci..

[37] López-Muñoz B., Greco Bermúdez L., Marín-Jiménez D., de la Fuente M.F.S., Capparelli A.F., Martínez I.M., Corredor S.S. (2019). An Unusual Amyloid Goiter in a 48-Year-Old Woman with Rheumatoid Arthritis, Secondary Amyloidosis and Renal Failure. Case Rep. Endocrinol..

[38] Stanaway A., Lam T. (2019). Consecutive cases of thyrolipomatosis and thymolipoma: A case report. ANZ J. Surg..

[39] Ravinder K., Abhishek B., Gagan J. (2019). Thyrolipomatosis: A Rare Fat Containing Lesion diffusely Infiltrating Throughout the Thyroid Gland. J. Assoc. Physicians India.

[40] Aggarwal A., Goyal A., Kandasamy D. (2020). Diffuse Thyroid Lipomatosis—A Rare Image. Indian J. Surg..

[41] Cavaco D.R., Alves Rafael A., Cabrera R., Vilar H., Leite V. (2021). Case Report: A Rare Association of Diffuse Thyroid Lipomatosis with Amyloid Deposition. Eur. Thyroid. J..

[42] Martí-Fernández R., Cassinello-Fernández N., Palomares-Casasús S., Gómez-Adrián J.C., Ferrández-Izquierdo A. (2021). Diffuse Lipomatosis of the Thyroid Gland. Am. Surg..

[43] Ayadi S., Hammami B., Boudaouara O., Boudawara T., Kallel S., Charfeddine I. (2021). [Association of thyrolipoma and thyrolipomatosis: A case report]. Ann. Pathol..

[44] Campion T., Maity A., Ali S., Richards P., Adams A. (2021). Concurrent thyrolipomatosis and thymolipoma in a patient with myasthenia gravis: A case report and review of the literature. Ann. R. Coll. Surg. Engl..

[45] Xhemalaj D., Xhardo E., Gradica F., Lisha L. (2022). Diffuse Lipomatosis of Thyroid Gland. Case Report and Review of Literature. Diagn. Pathol..

[46] Morado da Silva E.M., Ferreira R.A.d.C., Lozada A.R.C., Duarte J.M.S. (2022). A 54-Year-Old Woman with Papillary Thyroid Carcinoma Associated with Secondary Amyloid Goiter and Thyroid Lipomatosis. Am. J. Case Rep..

[47] Kesici U., Karatepe Y.K., Isceviren B. (2022). Concurrence of Thyrolipoma-tosis with Hyperthyroidism and Ectopic Thyroid Tissue. J. Coll. Physicians Surg. Pak..

[48] Kawai C., Miyao M., Kotani H., Minami H., Abiru H., Hamayasu H., Yamamoto A., Tamaki K. (2023). Systemic amyloidosis with amyloid goiter: An autopsy report. Leg. Med..

[49] Paz-Ibarra J., Concepción-Zavaleta M., Mendoza-Quispe D., Suárez-Rojas J., del Sur L.U.C., Fabián K.R., Deutz-Gómez D., Quiroz-Aldave J., Peralta J.S., Arróspide T.A. (2023). Coexistence of thyrolipomatosis and tongue squamous cell carcinoma: A case report. touchREVIEWS Endocrinol..

[50] Alenezi S., Saleem A., Alhajri O., Alozairi O. (2023). Thyrolipoma presentation as a huge multinodular goiter; A case report of an extremely rare entity. Int. J. Surg. Case Rep..

[51] Gonzalez-Gil A.M., Ruiz-Santillan M.A., Force B.K., Gaba R. (2024). A Case of Diffuse Thyroid Lipomatosis with Amyloid Deposits Presenting with Thyrotoxicosis. JCEM Case Rep..

[52] George D.M., Shah S.N. (2024). Diffuse Thyroid Lipomatosis and Amyloid Goiter With Incidental Papillary Thyroid Carcinoma: A Rare Case Report. Cureus.

[53] Willis R.A. (1958). The Borderland of Embryology and Pathology.

[54] Schroder S., Boeker W. (1985). Lipomatous Lesions of the Thyroid Gland: A Review. Appl. Pathol..

[55] Trites A.E.W. (1966). Thyrolipoma Thyrolipoma, Thymolipoma and Pharyngeal Lipoma: A Syndrome. Canad Med. Ass J..

[56] Gellhorn A., Marks P.A. (1961). The composition and biosynthesis of lipids in human adipose tissues. J. Clin. Investig..

[57] Derienzo D., Truong L. (1989). Thyroid Neoplasms Containing Mature Fat: A Report of Two Cases and Review of the Literature. Mod. Pathol..

[58] Lee M.H., Lee J.U., Joung K.H., Kim Y.K., Ryu M.J., Lee S.E., Kim S.J., Chung H.K., Choi M.J., Chang J.Y. (2015). Thyroid dysfunction associated with follicular cell steatosis in obese male mice and humans. Endocrinology.

[59] Basolo A., Poma A.M., Giannini R., Ceccarini G., Pelosini C., Fierabracci P., Castany M.U., Genzano S.B., Ambrosini C.E., Materazzi G. (2022). Histological pattern and gene expression profiling of thyroid tissue in subjects with obesity. J. Endocrinol. Investig..

[60] Martyniak K., Masternak M.M. (2017). Changes in adipose tissue cellular composition during obesity and aging as a cause of metabolic dysregulation. Exp. Gerontol..

[61] Gillmore J.D., Hawkins P.N. (2013). Pathophysiology and treatment of systemic amyloidosis. Nat. Rev. Nephrol..

[62] Villa F., Dionigi G., Tanda M.L., Rovera F., Boni L. (2008). Amyloid goiter. Int. J. Surg..

[63] Sung Y.N., Kim D., Kim J. (2022). p53 immunostaining pattern is a useful surrogate marker for TP53 gene mutations. Diagn. Pathol..

[64] Kim S., Cho S.W., Min H.S., Kim K.M., Yeom G.J., Kim E.Y., Lee K.E., Yun Y.G., Park D.J., Park Y.J. (2013). The Expression of Tumor-Associated Macrophages in Papillary Thyroid Carcinoma. Endocrinol. Metab..

[65] Cheung C.C., Ezzat S., Freeman J.L., Rosen I.B., Asa S.L. (2001). Immunohistochemical Diagnosis of Papillary Thyroid Carcinoma. Mod. Pathol..

[66] Hahn S.Y., Shin J.H. (2016). Description and comparison of the sonographic characteristics of poorly differentiated thyroid carcinoma and anaplastic thyroid carcinoma. J. Ultrasound Med..

[67] Hennessey J.V. (2011). Riedel’s thyroiditis: A clinical review. J. Clin. Endocrinol. Metab..

[68] Zala A., Berhane T., Christofer Juhlin C., Calissendorff J., Falhammar H. (2020). Riedel thyroiditis. J. Clin. Endocrinol. Metab..

[69] Kuk M., Kuo C.J., Nguyen V.H., Chen C.C. (2021). Synchronous thyrolipoma and papillary thyroid carcinoma: A rare but significant event. Diagnostics.

